# Long-Term Oral Treatment with Non-Hypoglycemic Dose of Glibenclamide Reduces Diabetic Retinopathy Damage in the Goto-KakizakiRat Model

**DOI:** 10.3390/pharmaceutics13071095

**Published:** 2021-07-17

**Authors:** Marianne Berdugo, Kimberley Delaunay, Cécile Lebon, Marie-Christine Naud, Lolita Radet, Léa Zennaro, Emilie Picard, Alejandra Daruich, Jacques Beltrand, Elsa Kermorvant-Duchemin, Michel Polak, Patricia Crisanti, Francine F. Behar-Cohen

**Affiliations:** 1 Physiopathology of Ocular Diseases: Therapeutic Innovations, Sorbonne University and Universityof Paris, Inserm UMRS 1138, F-75006 Paris, France; marianne.berdugo@gmail.com (M.B.); kimberley.delaunay@live.fr (K.D.); cecile.lebon@sorbonne-universite.fr (C.L.); marie-christine.naud@crc.jussieu.fr (M.-C.N.); lolitaradet@gmail.com (L.R.); lea.zennaro@inovarion.com (L.Z.); emilie.picard@crc.jussieu.fr (E.P.); adaruich.matet@gmail.com (A.D.); elsa.kermorvant@aphp.fr (E.K.-D.); patricia.lassiaz@crc.jussieu.fr (P.C.); 2 Department of Ophthalmology, AP-HP Hospital University Necker-Sick Children, F-75015 Paris, France; 3 Department of Paediatric Endocrinology, Gynecology and Diabetology, AP-HP Hospital University Necker-Sick Children, F-75015 Paris, France; jacques.beltrand@aphp.fr (J.B.); michel.polak@aphp.fr (M.P.); 4 Faculté de Santé, University of Paris, F-75006 Paris, France; 5 Institut Cochin, InsermU1016, F-75005 Paris, France; 6 Neonatal and Intensive Care Unit, AP-HP Hospital University Necker-Sick Children, F-75015 Paris, France; 7 Institute Imagine, InsermU1163, F-75015 Paris, France; 8 Ophthalmology, AP-HP Hospital Cochin, F-75005 Paris, France

**Keywords:** diabetic retinopathy, retinal neuroprotection, glibenclamide, glyburide, sulfonylureas, diabetes complications, retinal edema

## Abstract

Diabetic retinopathy (DR) remains a major cause of vision loss, due to macular edema, retinal ischemia and death of retinal neurons. We previously demonstrated that acute administration of glibenclamide into the vitreous, or given orally at a non-hypoglycemic dose, protected the structure and the function of the retina in three animal models that each mimic aspects of diabetic retinopathy in humans. In this pilot study, we investigated whether one year of chronic oral glibenclamide, in a non-hypoglycemic regimen (Amglidia^®^, 0.4 mg/kg, Ammtek/Nordic Pharma, 5 d/week), could alleviate the retinopathy that develops in the Goto-Kakizaki (GK) rat. In vivo, retinal function was assessed by electroretinography (ERG), retinal thickness by optical coherence tomography (OCT) and retinal perfusion by fluorescein and indocyanin green angiographies. The integrity of the retinal pigment epithelium (RPE) that constitutes the outer retinal barrier was evaluated by quantitative analysis of the RPE morphology on flat-mounted fundus ex vivo. Oral glibenclamide did not significantly reduce the Hb1Ac levels but still improved retinal function, as witnessed by the reduction in scotopic implicit times, limited diabetes-induced neuroretinal thickening and the extension of ischemic areas, and it improved the capillary coverage. These results indicate that low doses of oral glibenclamide could still be beneficial for the prevention of type 2 diabetic retinopathy. Whether the retinas ofpatients treated specifically with glibenclamideare less at risk of developing diabetic complications remains to be demonstrated.

## 1. Introduction

Diabetic retinopathy remains a major cause of vision loss in the working-age population of Western countries, andthe incidence may increase due to the increased prevalence of diabetes worldwide [1]. Indeed, despite the widespread use of intraocular anti-VEGF drugs and glucocorticoids, mainly designed to act on macular edema, visual function is threatened by ischemic insults and by direct diabetic-induced neuropathy [2,3]. The neuropathy occurs early, even before microangiopathy is detected, as shown by recent retinal imaging technologies [3] or by electrophysiology showing progressive deterioration of electrical retinal responses with loss of electrical signal amplitudes and extended response times, reflecting retinal neuron alterations [4,5]. This could explain the fact that despite long-term repeated intraocular injections of anti-VEGF and glucocorticoids, visual gain remains limited, of around five letters in real world studies [6,7]. To date, there is no available treatment to stop or even slow down retinal neurodegeneration.

Sulfonylureas, through binding to the sulfonylurea receptor (SUR), which is a subunit of SUR1-Kir6.2 and SUR1-TRPM4(NCCa-ATP) channels, not only provoke insulin secretion by the pancreas [8], but also exert neuroprotective effects through binding to SUR1 expressed in neurons, astrocytes, oligodendrocytes, endothelial cells and reactive microglia [9,10,11,12,13]. In cerebral ischemia, glibenclamide reduced brain edema, was neuroprotectant [14], and decreased the risk of haemorrhagic transformation after severe ischemia [13]. Recently, in a model oftemporary middle cerebral artery occlusion, low doses of oral glimepiride improved neurological functions, reduced infarct volume and brain edema, restored tight junction protein expression and suppressed inflammatory cytokines [15]. Large meta analysis have recapitulated results on the protective effect of oral sulfonylurea drugs and tried to define their therapeutic potential in stroke [16]. In the context of diabetes, children presenting a potassium channel mutation responsible for diabetes showed improved neuropsychological development after they were treated specifically with glibenclamide (glyburide) [17,18]. In patients with type 2 diabetes, large meta analysis showed that sulfonylureas, similarly to metformin, but unlike insulin therapy, improved the cognitive dysfunction [19].

In the field of ophthalmology, Nicholson et al. showed that although glibenclamide did not protect the optic nerve’s proximal microvasculaturein a rodent model of anterior ischemic optic neuropathy, it reduced the optic nerve head edema [20]. We recently showedthat local delivery of glibenclamide protected against excitotoxic stress in vivo and improved the function and the structure of the diabetic retina in a type 2 diabetic rat model [21]. Indeed, the direct intraocular injection of glibenclamide in a model of excitotoxic retinal cell death increased a and b-wave ERG amplitudes and, in the Goto-Kakizaki diabetic rat, it reduced the b-wave ERG implicit times, demonstrating its ability to improve retinal function. In a neonate rat model of hyperglycemia-induced retinal degeneration, glibenclamide prevented ganglion cells loss. In addition, we showed that SUR1-TRPM4 and SUR1-Kir_6.2_ co-localized in the primate macula that is enriched in cone photoreceptors, showing a potential direct effect of glibenclamide on photoreceptors [21]. This observation is supported by a recent study showing that glibenclamide regulates photoreceptor metabolism and inhibits the production of oxygen reactive species induced by the exposure of the photoreceptor’s outer segment to light [22]. Altogether, these results indicate that sulfonylureas could exert direct neuroprotective effects on the diabetes-induced retinal neurodegeneration. To answer this question, we treated type 2 diabetic Goto-Kakizaki (GK) rats orally with a non-hypoglycemic dose of glibenclamide for 10 months and evaluated its effect on retinal function and diabetic retinopathy signs.

In summary, diabetic retinopathy remains one of the major causes of visual loss due to macular edema, ischemia and neuronal cell loss. Sulfonylureas, through binding to their receptor SUR1, show neuroprotective effects in the central nervous system as well as in rodent models of excitotoxicity and hyperglycemia-induced retinal degeneration. Short-term treatment with glibenclamide protected the ERG-assessed retinal function and reduces retinal cell death [21]. Moreover, SUR1 is expressed in the macula, particularly in cones that are responsible for visual acuity and colour vision, suggesting that glibenclamide might protect the diabetic retina in humans. In the present study, we used in vivo methods to evaluate whether long-term oral treatment with glibenclamide at a non-hypoglycemic dose could preserve the function and the morphology of GK rats’ retinas.

## 2. Material and Methods

### 2.1. Animals and Animal Model; Ethical Concerns

The Goto-Kakizaki rat is a non-obese and non-insulin-dependent type 2 diabetes mellitus model. It was raised in our animal facility from Taconic’s genitors (Silkeborg, Denmark). HbA1c was measured with A1C NOW+ multitest system (Bayer, Leverkusen, Germany) at 2, 6 and 12 months. Diabetes was defined as a plasma glucose level >250 mg/dL (14 mmol/L). In our colony, and as previously observed, only males developed hyperglycemia at approximately 8 weeks of age that stabilized around 14 weeks of age [23]. Metabolic dysfunction in the GK rat involves three pathogenic events with complex interactions: (1) three independent mutations in loci involved in insulin secretion, (2) gestational hyperglycemia responsible for a transgenerational decrease in the beta-cell mass, (3) loss of beta-cell differentiation due to chronic exposure to hyperglycemia [24]. Male GK rats develop late complications such as microangiopathy, nephropathy, neuropathy and retinopathy [25]. Although no animal model recapitulates all the features of human diabetic retinopathy, male GK rats develop progressive retinopathy with local inflammation, retinal circulatory abnormalities [26] and retinal edema [26,27,28,29], together with a thickening of the choroid [27], before microangiopathy develops at around 7 months [30], mimicking the kinetics of the events in human diabetic retina [31]. The retinopathy involves a PKCzetaoveractivation-linked blood–retinal barrier (BRB) breakdown, photoreceptor neurodegeneration [28,32], Müller cell swelling and morphological damage of the outer retina [21]. Our present study extended from 2 months up to 12 months of age, i.e.,from before high blood glucose onset until the advanced stage of retinopathy in male GK rats (*n* = 10 eyes in the glibenclamide-treated group; *n* = 18 eyes in the vehicle-treated group). We used female euglycemic GK rats (*n* = 10 eyes) and Wistar rats (*n* = 12 eyes) as controls. Wistar rats were chosen as controls for ERG, since one study reported in 2019 that although no diabetes develops in female GK rats, some ERG abnormalities might occur [33].

All rats were kept in pathogen-free conditions with ad libitum food and water and were housed in a 12 h light/12 h dark cycle. They were weighed once a week. During the study, one rat died in the vehicle-treated and one in the glibenclamide-treated diabetic groups.

All experimental procedures were performed in accordance with the Association for Research in Vision and Ophthalmology (ARVO) statement for the use of animals in Ophthalmic and Vision Research as well as with the 2010/63/EU UE directive. Experimental procedures comply with the 3Rs and were approved by the local ethics committee: Charles Darwin European Council of the Université de Paris (authorization 03952.03, A75-580/A750612). All procedures were performed under general (ketamine 40 mg/kg, Kétamine 1000^®^, Virbac, France + xylasine 4 mg/kg, Rompun 2%^®^, Bayer AG, Leverkusen, Germany) and local anesthesia (oxybuprocainchlorhydrate, Tetracaine^®^Théa Pharma, Clermont-Ferrand, France). Animals were sacrificed by intraperitoneal sodium pentobarbital (150 mg/kg) prior to enucleation.

### 2.2. Long-Term Oral Glibenclamide Treatment

Male GK rats were treated from 2 to 12 months with either oral pediatric glibenclamide suspension (Amglidia^®^ 6 mg/mL, 0.2 mg/kg BID, Ammtek/Nordic Pharma, Paris, France—glibenclamide in hydroxyethylcellulose, lactic acid, purified water, sodium benzoate (E211), sodium citrate, xanthan gum) or with the vehicle. Non-diabetic control rats were treated by the same volume of vehicle. Oral gavage was performed twice a day (8:00 a.m. and 6:00 p.m.), 5 days a week. This drug regimen, not comparable to human treatment for diabetes, was chosen to induce minimal changes in the glycemic control. Since the half-life of glibenclamide is 8–10 h, drug levels are expected to be maintained after the drug is stopped and until it is eliminated (i.e., 2.3 days, covering the week end).

### 2.3. qPCR–ABCC8 Neuroretinal Expression

At 12 months, at the end of the treatment period, rats were sacrificed and neuroretinas from 5 vehicle-treated diabetic retinas and 3 glibenclamide-treated ones were isolated on ice and directly frozen until RNA isolation. Total RNA was extracted using the RNeasy mini kit (Qiagen, Courtaboeuf, France) according to the manufacturer’s protocols. RNA concentration, purity and integrity were determined with a NanoPhotometer (Implen, München, Germany). First-strand cDNA was generated by reverse transcription using 0.5 μg total RNA and the II Reverse TranscriptaseKit (Invitrogen, Waltham, MA, USA). Genomic DNA was removed on RNeasy columns before reverse transcription, according to the manufacturer’s protocols. Expression of the ABCC8 gene, encoding the SUR1 receptor, and the GAPDH housekeeping gene was evaluated in a 96-well plate with Quantstudio 5 (Applied Biosystems by Thermofisher scientific, Waltham, MA, USA). Each PCR reaction was performed following manufacturer’s instructions. The primers used are detailed in Table 1. The threshold for calculating cycle threshold (C_t_) values was calculated automatically using QuantStudio 3 and 5 Real-Time PCR System Software.The relative expression of each gene was calculated using the 2-ΔΔCt method. Ct values were analyzed with the fold change and *p* value of each gene (Student’s *t*-test).

### 2.4. Electroretinography (ERG)—Visual Function Evaluation

Bilateral full-field ERG responses were recorded at 12 months of age, following a 10-month-long treatment of either glibenclamide or vehicle oral suspension. Rats were anaesthetized by intramuscular ketamine (80 mg/kg)/xylazine (8 mg/kg). The cornea was desensitized with a drop of 1% tetracainchlorhydrate (Tétracaine^®^, Théa Pharma, Clermont-Ferrand, France), and mydriasis was obtained with drops of 0.5% tropicamid (Mydriaticum^®^, Théa Pharma, Clermont-Ferrand, France). Eyelids were kept open by home-made latex “surgical drapes”. Flash ERG recordings were performed with the GanzfeldVisioSystem device (Siem Bio-Médicale, Nîmes, France), adapted for rodents, sending light flashes. For scotopic electroretinograms, essentially representing rod-driven responses, rats were dark-adapted overnight. Flash intensities ranged from 0.0003 up to 10 cd.s/m^2^. For cone-driven responses, photopic electroretinograms were recorded following a 5 min light adaptation and a 10 cd.s/m^2^ flash intensity. In both conditions, 5 responses were averaged. Mixed responses refer to whole retinal responses (coming from both cone and rod-driven pathways). Negative a-wave amplitudes were measured from baseline to trough bottom. Positive b-wave amplitudes were measured from the bottom of the b-wave’s trough to its peak. Implicit times of the first two oscillatory potentials were measured from time of stimulus to peaks. Results were expressed in microvolts (µV) for amplitudes and milliseconds (ms) for implicit times (mean ± SD) (*n* = 7 to 16 eyes per group). Cursors were placed in a blind way. For details, see [21] Suppl Data.

### 2.5. Fundoscopy, Optical Coherence Tomography (OCT)—Neuroretinal Thickness Measurement

In vivo imaging of rat retinas was performed on anesthetized animals using the Micron III high resolution retinal imaging microscope (Phoenix Technology Group, Pleasanton, CA, USA) adapted for small animal eyes. Funduscopic examinations and Optical Coherence Tomography were performed at 12 months. OCT is a non-invasive imaging method that provides retina sections along chosen lines on fundus images. At least 5 OCT images were taken for each eye (one central and one temporal, nasal, inferior and superior quadrants of the retina), using the optic nerve head as a landmark. Neuroretinal thickness, measured with ImageJ software 3 times in a masked fashion, was compared in the same direction and same distance from the optic nerve.

### 2.6. Fluorescein and Indocyanin Green Angiographies—Retinal and Choroidal Vascularization Imaging and Evaluation of Dye Vascular Infusion

In vivo fluorescein and indocyanin green (ICG) angiographies were simultaneously performed to obtain images of both retinal and choroidal structures, in all 12-month-old rats. In this process, 150 μL of a mixture made of fluorescein (0.5% Fluorescein^®^ Faure for intravenous injections) and ICG (Infracyanin, 0.5 mg in 5% glucose; Serb) (50:50, *v*/*v*) wasinjected in the tail vein of anesthetized rats. Angiography pictures were taken with a confocal scanning laser ophthalmoscope (Spectralis Heidelberg Retinal Angiograph, HRA2, Heidelberg Engineering/Sanotek, Heidelberg, Germany) using a blue laser (488 nm, barrier filter at 500 nm) and an infrared laser (795 nm, barrier filter at 800 nm) for dye excitation. Bilateral images were collected at various time points.

Using a subjective scale and using all time points of angiography pictures, we scored the infusion of dyes within the retinal vessels in a blind fashion, as follows:

3—All vessels normally infused with fluorescein, no ischemic area

2—Some vessels are not well-infused, or few ischemic areas appear

1—Most retinal vessels are not well-infused with fluorescein and/or ischemic areas are present

0—Fluorescein infusion modified in the whole retina and/or many ischemic areas

We also counted the total number of ischemic areas per eye.

### 2.7. Flatmounts, Immunohistochemistry and Home-Made Macro Analysis for Evaluation of Capillary Percentage among Retinal Vessels and RPE cell Areas Analysis

After sacrifice, the eyes were enucleated, fixed for 1 h in 4% paraformaldehyde. They were dissected and the neuroretinas separated from RPE/choroid/sclera complex. Four radial cuts were made in each tissue for flat-mounting fixed 10 min at −20 °C in acetone, blocked with fetal bovine serum 10% in PBS, 0.1% Triton for 30 min. Retinas (*n* = 3 to 6/group) were then incubated overnight at 4°C with TRITC-conjugated lectin (Sigma-Aldrich, St. Louis, MI, USA) at 1:300, 5 min in Dapi (4’,6-diamidino-2-phénylindole) 1:5000 at RT, and flat-mounted with Fluoromount mounting medium (Sigma-Aldrich, St. Louis, MI, USA). RPE/choroid complexes (*n* = 3 to 6/group) were incubated with rhodamine-conjugated anti-phalloidin antibody (Invitrogen, Waltham, MA, USA) 1:300 overnight at 4 °C. Negative controls consisted of the omission of lectin or conjugated antibody. Six pictures per neuroretina at ×8 magnification, and 20 pictures per RPE/choroid/sclera complex at ×40 magnification, distributed all over the entire surface, were acquired with an Olympus microscope (BX51). Using these images and ImageJ software with a macro tool [29] developed on Fiji software, RPE images were used for morphological analysis, including RPE cell areas, as described in [29].

### 2.8. Statistics

Data are provided as mean ± SD in non-diabetic, diabetic, and glibenclamide-treated diabetic groups at 6 or 12 months (i.e., at the termination of long-term treatment). Statistical analysis was performed using Graph Pad Prism 8. ANOVA followed by multiple comparison post-tests was used when more than two groups were compared (exact *p* values are given), and theMann–Whitney-U test was used to compare two groups. *p* < 0.05 was considered significant.

## 3. Results

### 3.1. Long-Term Oral Glibenclamide at 200 µg/kg BID did Not Influence Body Weight and Glycemic Control in GK Rats–qPCR ABCC8 Expression

**Figure 1:**
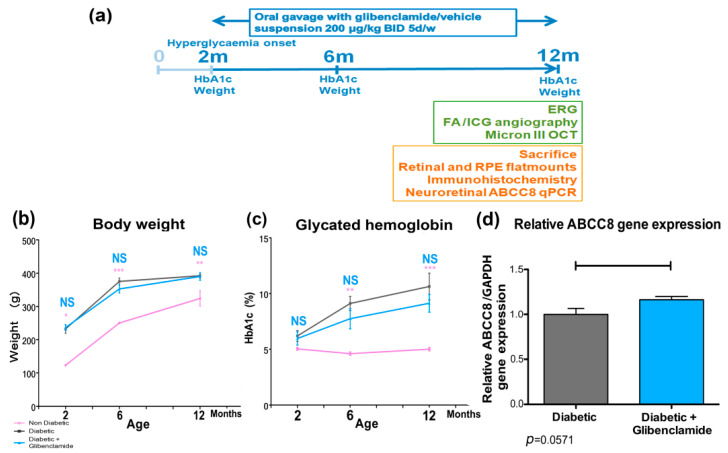
Schematic timeline schedule of the experiment, body weight, glycated hemoglobin, and relative ABCC8 (SUR1) qPCR expression. (**a**) Schematic timeline schedule of the experiment. (**b**) Body weight expressed as mean ± SEM at 2, 6 and 12 months. ANOVA and Bonferroni’s post-test vs. diabetic. * *p* < 0.05; ** *p* < 0.01; *** *p* < 0.001 (*n* = 5, 8 and 5 rats, respectively, at 2 months, *n* = 3, 8 and 5 rats at 6 months, and *n* = 3, 8 and 4 rats at 12 months). Non-diabetic = female GK rats; (**c**) glycated hemoglobin (HbA1c) in non-diabetic, diabetic and glibenclamide-treated diabetic animals, expressed as a mean percentage ± SEM at 2, 6 and 12 months (*n* = 3, 8 and 5 rats, respectively, at 2 months, *n* = 3, 8 and 5 rats at 6 months, and *n* = 3, 8 and 4 rats at 12 months). ANOVA and Bonferroni’s post-test vs. diabetic. ** *p* < 0.01; *** *p* < 0.001; (**d**) Relative qPCR ABCC8 gene expression in neuroretinas at 12 months (after long-term low-dose oral glibenclamide treatment; *n* = 5 and 3 rats, respectively, in diabetic and glibenclamide-treated diabetic animals). Mann–Whitney test *p* = 0.057. Non-diabetic control = pink, diabetic = grey, long-term glibenclamide-treated diabetic = blue.

Neither body weight nor glycated hemoglobin differed between the vehicle and glibenclamide-treated diabetic groups of male Goto-Kakizaki rats at 2, 6 and 12 months of age (Figure 1 and Table 2; two-way ANOVA and Bonferroni post-test; * *p* < 0.05, ** *p* < 0.01, *** *p* < 0.001 significant only for non-diabetic females vs. diabetic males GK rats). Quantitative PCR expression of ABCC8 gene mRNA normalized with GAPDH mRNA tends to be increased by treatment in 12-month-old diabetic rat neuroretinas (*p* = 0.057).

### 3.2. Long-Term Non-Hypoglycemic Oral Glibenclamide Treatment Improves Retinal Function in GK Rats

**Figure 2:**
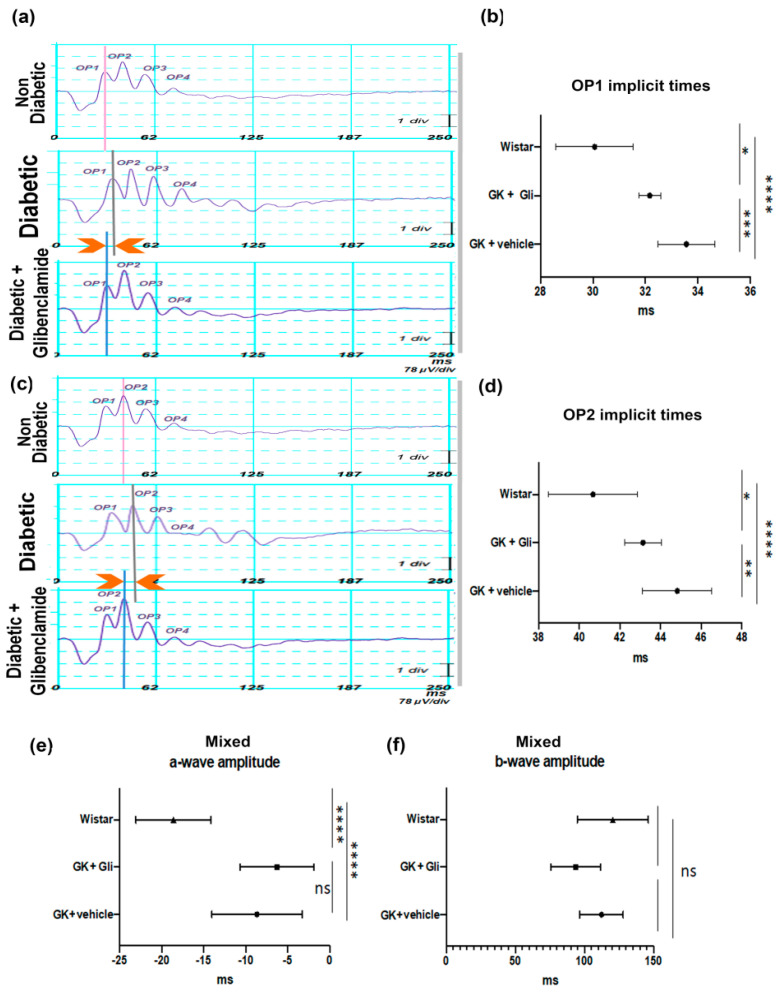
Long-term low-dose oral glibenclamide treatment improves retinal function through a reduction in ERG implicit times in diabetic Goto-Kakizaki rats. (**a**,**c**) Representative scotopic oscillatory potentials charts recorded in 12-month old non-diabetic Wistar rats (*n* = 16 eyes), vehicle-treated (*n* = 17 eyes) or glibenclamide-treated (*n* = 7 eyes) diabetic GK rats, showing an extension of implicit times with diabetes and a reduction with treatment; (**b**) OP1 (first oscillatory potential) (* *p* < 0.05, *** *p* < 0.001, **** *p* < 0.0001) and (**d**) OP2 corresponding implicit time graphs (* *p* < 0.05, ** *p* < 0.01, **** *p* < 0.0001). (**e**) Mixed a-wave (**** *p* < 0.0001) and (**f**) mixed b-wave amplitude graphs in same groups. Mean ± SD, ANOVA and multiple comparison post-test.

At 12 months and after 10 months of oral treatment, ERG parameters that reflect the inner retinal activity were improved in male GK rats treated with glibenclamide as compared to untreated male GK, as shown by the significant reduction in implicit times (Figure 2). Indeed, the first and second scotopic oscillatory potential implicit times were, respectively 30.06 ± 1.48 and 40.7 ± 2.18 ms in non-diabetic Wistar rats (*n* = 16), 33.5 ± 1.09 and 44.8 ± 1.7 ms in vehicle-treated diabetic rats (*n* = 17) as compared to 32.1 ± 0.4 and 43.1 ± 0.9 ms in glibenclamide-treated diabetic rats(*n* = 7) (**** *p* < 0.0001, * *p* = 0.02 and *** *p* = 0.0009; **** *p* < 0.0001, * *p* = 0.04 and ** *p* = 0.005). Mixed a- and b-wave amplitudes were reduced by diabetes (−18.6 ± 4.47 µV vs. −8.67 ± 5.37 µV and 120.5 ± 25.59 µV vs. 112.3 ± 15.67 µV), and not significantly protected by treatment (−6.29 ± 4.38 µV and 93.7 ± 17.8 µV; **** *p* < 0.0001 and ns). In photopic ERG conditions (not shown), the mixed blue flashes’ a-wave implicit time that reflects the photoreceptors activity was significantly shorter in treated animals (mean implicit time 18.50 ± 2.7 ms vs. 24.61 ± 0.92 ms, *p* = 0.024). These results demonstrate improved electrical response times of the inner and the outer retina in diabetic GK rats treated by oral glibenclamide over a long period of time.

### 3.3. Long-Term Non-Hypoglycemic Oral Glibenclamide Treatment Reduces Retinal Edema in GK Rats

**Figure 3:**
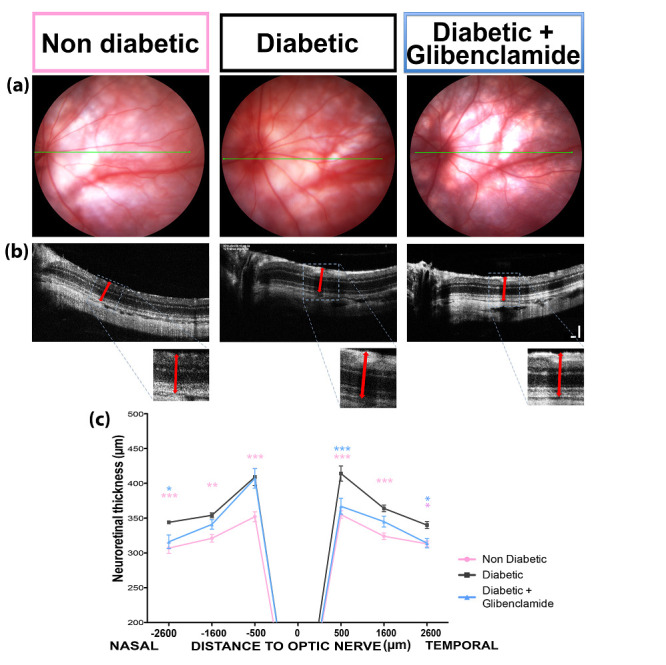
Long-term low-dose oral glibenclamide treatment reduces diabetes-induced retinal edema in diabetic Goto-Kakizaki rats. (**a**) Fundi pictures of 1-y-old non-diabetic female, diabetic and glibenclamide-treated diabetic rats obtained with Micron III device (Phoenix Research Labs); (**b**) corresponding Micron III OCT pictures used to measure neuroretina thickness at fixed distances from the optic nerve and known orientation (nasal-temporal); insert = thickness measurement; vertical scale bar = 200 μm, horizontal scale bar = 100 μm. (**c**) Neuroretinal thicknesses at 500, 1600 and 2600 μm from the optic nerve, in nasal and temporal directions, in non-diabetic Wistar and female GK (pink line), diabetic (black line) and glibenclamide-treated diabetic (blue line) rats (*n* = 6 to 20 eyes). Two-way ANOVA and Bonferroni’s post-test vs. diabetic group; * *p* < 0.05, ** *p* < 0.01, *** *p* < 0.001.

At the end of follow-up, OCT B-scans were taken in 12-month-old diabetic and glibenclamide-treated diabetic rats and in age-matched control rats (Figure 3). In male GK rats, the neuroretinal thickness was increased as compared to control rats (Figure 3c). Long-term oral glibenclamide partially prevented retinal thickening (Figure 3c), with a maximal effect observed in the temporal direction near the optic nerve (mean thicknesses were 349 ± 3 μm in non-diabetic animals, 414 ± 11 μm in diabetic rats and 367 ± 11 μm in glibenclamide-treated diabetic rats, two-way ANOVA *p* < 0.0001, Bonferroni post-test *p* < 0.001 vs. diabetic group). The thickness reduction was also significant at 2600 μm from the optic nerve in both nasal and temporal directions (mean thicknesses were, respectively, 304 ± 5 and 311 ± 3 μm in non-diabetic animals, 344 ± 2 μm and 340 ± 5 μm in diabetic rats and 316 ± 9 μm and 314 ± 7 μm in glibenclamide-treated diabetic rats, Bonferronipost test *p* < 0.05 vs. diabetic group). Note that the retinal thickness remains higher in glibenclamide-treated rats as compared to non-diabetic controls, without retinal thinning.

### 3.4. Long-Term Non-Hypoglycemic Oral Glibenclamide Improves Retinal Vascular Perfusion in Diabetic Goto-Kakizaki Rats

**Figure 4:**
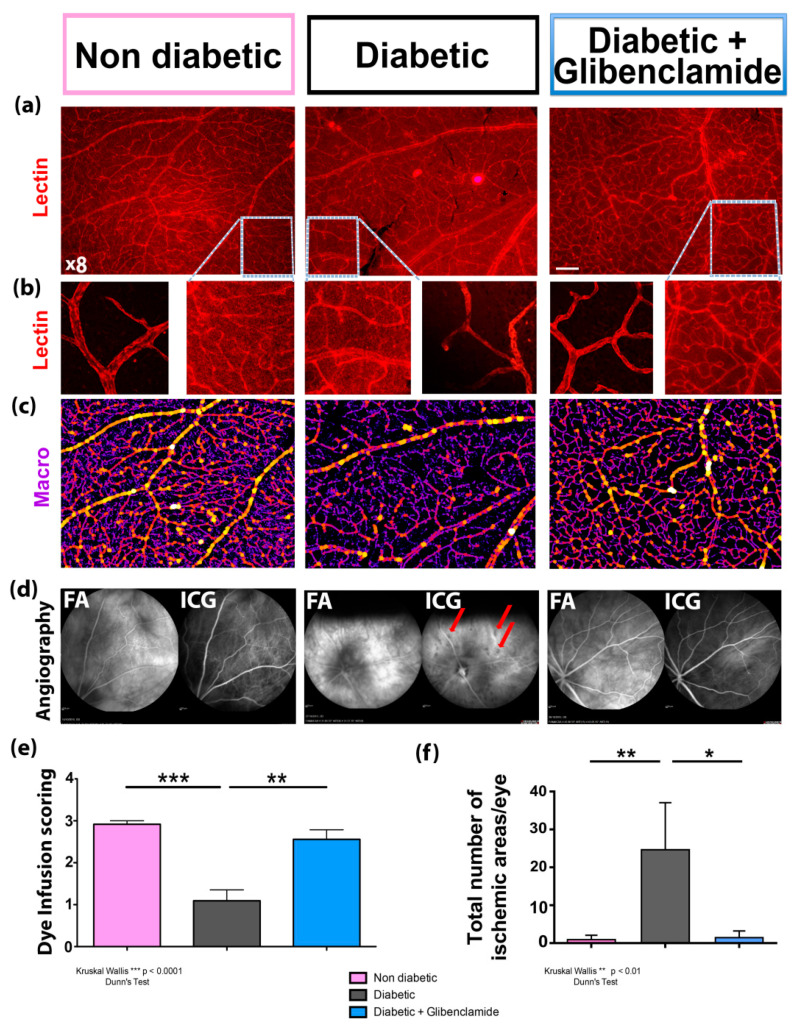
Glibenclamide improves retinochoroidal blood perfusion, and reduces the number of ischemic areas in diabetic Goto-Kakizaki rats. Non-diabetic female GK (left column) vs. diabetic male GK (middle column) vs. glibenclamide-treated diabetic GK (right column) rats. (**a**,**b**) Fluorescence microscope pictures of retinal flatmounts labeled with TRITC-lectin in 1-y old rats (×8 in (**a**) Bar = 200 µm; higher magnification in (**b**)); (**c**) analyses of the previous pictures with a home-made macro in ImageJ software, which detects retinal vascular network and classifies vessels according to their diameters (small vessels in purple, medium vessels in red and large vessels in yellow); (**d**) in vivo confocal fluorescein (FA, left) and indocyanin green (ICG, right) angiographies at 4.30 min post dye intravenous injection in same groups; non-perfused ischemic spots (red arrows). (**e**) Subjective scoring of dye infusion in vessels at all time points (ANOVA *p* < 0.0001 and multiple comparison Dunn’s post-test (** *p* < 0.01 and *** *p* < 0.001)in diabetic vs. non diabetic groups, and glibenclamide-treated vs. non-treated groups, *n* = 12, 16 and 9 eyes, respectively); (**f**) number of ischemic areas on angiographies in non-diabetic vs. diabetic vs. glibenclamide-treated diabetic groups (*n* = 10, 7 and 5 eyes, respectively). Kruskal–Wallis (*p* < 0.01) and multiple comparison Dunn’s post-test (* *p* < 0.05, ** *p* < 0.01). Non-diabetic wistar + female GK rats = pink bar; diabetic GK rats = grey bar; glibenclamide-treated diabetic GK rats = blue bar.

As shown in Figure 4e, vascular perfusion was significantly reduced in diabetic GK rats as compared to non-diabetic controls and glibenclamide significantly improved the perfusion angiographic score (ANOVA *p* < 0.0001 and multiple comparison Dunn’s post-test *p* < 0.001 and *p* < 0.01; *n* = 12, 16 and 9, respectively). Non-perfused areas were also significantly reduced in glibenclamide-treated diabetic rats as compared to untreated diabetic controls (Kruskal–Wallis *p* < 0.01 and Dunn’s post-test, *p* < 0.01 and *p* < 0.05; *n* = 10, 7 and 5, respectively) (Figure 4f). To further confirm the in vivoangiographic results, rats were sacrificed, and retinas were flat-mounted and labeled with TRITC-lectin (red), as shown in Figure 4a (and Figure 4b at higher magnifications). Glibenclamide improved the retina coverage with small capillaries as compared to untreated diabetic rats (43.6 vs. 35.8% of small capillaries among vessels, *p* = 0.04). These converging results indicate that a lowdose of glibenclamide may reduce retinal microvascular abnormalities associated with chronic diabetes.

### 3.5. Long-Term Non-Hypoglycemic Oral Glibenclamide Prevented Outer Retinal Barrier Damages

**Figure 5:**
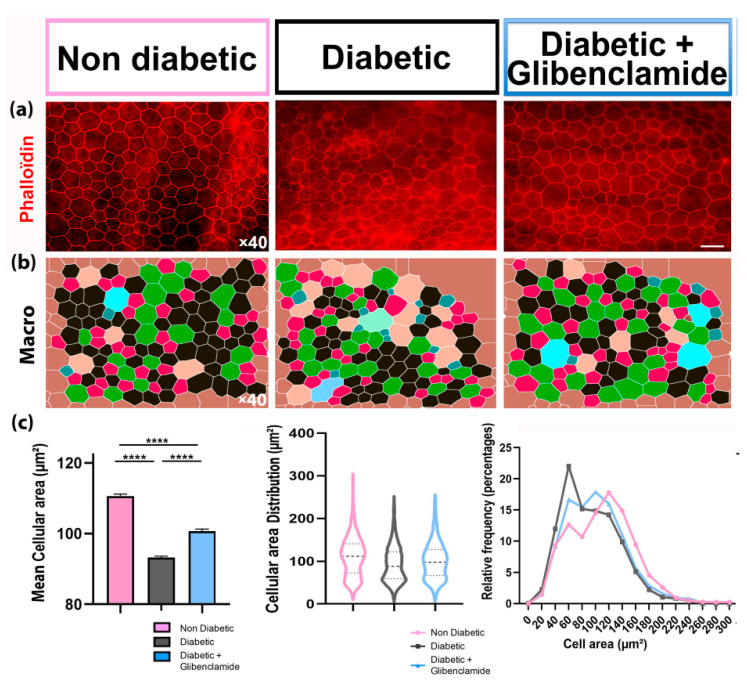
Long-term low-dose oral glibenclamide treatment protects RPE against diabetes-induced change in diabetic Goto-Kakizaki rats. (**a**) Retinal pigment epithelium flatmounts of 1-y-old non-diabetic female, diabetic and glibenclamide-treated diabetic rats; immunohistochemistry with anti-phalloïdin antibody and fluorescence microscope pictures (×40); bar = 40 µm; (**b**) morphological analysis of those RPE pictures with a home-made macro in ImageJ software; (**c**) mean RPE cellular area (μm^2^) ± SEM (left; ANOVA *p* < 0.0001 followed by Dunn’s post-test **** *p* < 0.0001; *n* = 5983, 10,294 and 6232 cells of 7, 4 and 4 eyes, respectively); distribution of cell areas (middle); relative frequency of cell areas (right) in each group: non diabetic control wistar + female GK rats = pink bar, violin and line; diabetic GK rats = grey bar, violin and line; glibenclamide-treated diabetic GK rats = blue bar, violin and line.

On RPE/choroid flatmounts, RPE cells from male GK rats, stained with anti-phalloidin antibody (Figure 5a) exhibit increased cellular polydispersity and reduced cell size [29]. Indeed, in old diabetic rats, the mean RPE cellular area, smaller than in non-diabetic controls, was partially restored by glibenclamide (mean area = 93.2 ± 0.6 μm^2^ vs. 110.6 ± 0.4 μm^2^, Kruskal–Wallis test *p* < 0.0001 and Dunn’s multiple comparison test, *p* < 0.0001 in non-diabetic vs. diabetic groups; 100.7 ± 0.5 μm^2^, Dunn’s multiple comparison test, *p* < 0.0001 in glibenclamide-treated vs. vehicle-treated diabetic groups). Glibenclamide also reduced RPE cells’ polydispersity induced by diabetes (Figure 5). The effect of long term oral glibenclamide on RPE morphology changes indicates that glibenclamide could also prevent outer retinal barrier dysfunction.

## 4. Discussion

We previously showed that short-term ocular administration of glibenclamide in the rat vitreous efficiently reduced retinal cell death in excitotoxic models and protected the retina of diabetic GK rats [21], confirming that glibenclamide is also neuroprotective in the retina. Interestingly, SUR1 and TRPM4 as well as Kir6.2 are expressed in glial retinal cells, ganglion cells, interneurons and in photoreceptors, particularly in primate photoreceptors in the macula, suggesting that glibenclamide can act directly on retinal cells and not only on the neurovascular unit. Recently, Ravera et al. used in vitro experiments to show that glibenclamide could interfere with the respiratory complex of rods by targeting the mitochondrial respiratory complexes I, II, and III and inhibiting the oxidative metabolism and oxidative stress. They also showed a synergistic effect of glibenclamide with metformin on reducing the production of oxygen reactive species [22].

Our study confirms that glibenclamide exerts beneficial effects on various signs of diabetic retinopathy in the GK rats, even when taken orally at a non-hypoglycemic dose for a long period of time.

On ERG, the scotopic oscillatory potentials resulting from the activity of the ON-bipolar cells and the lateral inhibitory pathways of the inner retinal neurons [34] are delayed in diabetic vs. non-diabetic animals, as shown by the extension of their implicit times. Glibenclamide significantly reduced the implicit times of scotopic oscillatory potentials (that mostly measures rod pathway function). This is in line with our previous results showing that short-term glibenclamide treatment reduces scotopic b-wave implicit time delay in old GK rats [21]. Similarly, OPS delay was observed in rats with streptozotocin-induced diabetes [35,36] and in patients with type 2-diabetes with or without retinopathy [35]. In patients with diabetes, the OP1 implicit time delay is recognized as an early sign of visual dysfunction, whilst other OPS implicit time delays occur later [5]. On the other hand, an oral low dose of glibenclamide did not prevent the diabetes-induced a and b-wave amplitude reduction, suggesting that further studies remain to be conducted in other models of diabetes, to determine the optimized dose and route of glibenclamide administration to prevent efficiently retinopathy.

Retinal thickness, measured more specifically in the macula, is the main clinical parameter to detect and quantify edema and guide therapeutic indications in diabetic patients. Although no rodent model can recapitulate diabetic macular edema, similar pathogenic mechanisms have been described in GK rats and in humans [37], and in diabetic GK rats, retinal thickness is also increased but in a more diffuse manner. Using a long-term low dose of glibenclamide, retinal thickness was reduced, as observed with short-term local treatment [21], although the retina remained thicker than in non-diabetic Wistar rats and no thinning was observed. Depending on the model and the studied time points, various retinal morphological changes were reported in vivo using OCT. Whilst retina thinning was observed in 20-week-old db/+ mice [38], retina thickening was noted in 40 week-old diabetic Torii fatty and non-fatty rats [39], showing that in the long term, loss of visual neurons can be measured.

The anti-edematous effect of glibenclamide has been well documented in the brain under ischemic conditions [40] and a large multicentric randomized controlled study is currently evaluating the potential beneficial effects of glibenclamide for cerebral edema reduction after intracerebral hemorrhage [41]. In ischemic or traumatic brain cells, binding of glibenclamide to SUR1-TRPM4 reduces depolarization, which reduces blood–brain barrier breakdown and vasogenic edema [42]. In addition, in the retina, glibenclamide anti-edematous effects could result from the inhibition of the NOD-like receptor pyrin domain containing 3 (NLRP3) inflammasome and related neuroinflammation [43], known to be involved in diabetic retinopathy mechanisms [44,45,46]. In clinical studies, whether glibenclamide or other sulfonylurea drugs could improve the management of patients with diabetic macular edema has not been demonstrated, but the present result could prompt further studies on the subject.

Finally, a low dose of oral glibenclamide reduced the retinal microvasculature abnormalities detected in one-year-old male GK rats and protected RPE cells, which form the outer retinal barrier. These results show that glibenclamide, even at a very low dose, exerts beneficial effects on the different mechanisms causing vision loss in patients.

The exact dose that reaches the eye after oral administration of glibenclamide at 400 μg/kg/d cannot be ascertained, although previous studies showed that efficient drug levels can be reached in the brain even at much lower doses (i.e., 4 µg/kg/d). In a previous study, we showed that one oral administration of 210 µg/kg, which is a similar dose, of glibenclamide suspension (Amglidia^®^, Ammtek/Nordic Pharma, France) in 9-week-old Wistar rats resulted in 0.26 and 0.3 ng/mL intraocular concentrations 1 and 2 h after intake, respectively, which were sufficient to achieve a neuroprotective effect locally [21]. In diabetic animals, we expect the intraocular biodisponibility to be higher, due to the breakdown of blood retinal barrier. It should be noted that the doses used to lower blood glucose in the GK rat (1 to 50 mg/kg) are higher than in humans [47,48,49,50].

In clinical practice, diabetic patients are treated for years with doses of glibenclamide up to 15 mg/day, and it is thus expected that neuroprotective doses will be reached in the retina, especially with regard to the barrier breakdown that occurs. Other common hypoglycemic drug classes have shown neuroprotective effects in the brain and in the retina, such as metformin, with potential synergistic effects with glibenclamide. On the other hand, insulinotherapy has been associated with reduced cognitive functions in patients with moderate type 2 diabetes [19].

A possible limitation of this study is a non-statistically significant trend toward a decrease in glycated hemoglobin in the glibenclamide-treated group, at 400 μg/kg/d orally. However, in our previous experiments, glibenclamide was administered (1) orally, at the same dosing regimen, in streptozotocin-induced hyperglycemic rat pups, and (2) intravitreously in adult rats, and in both conditions, although it did not influence HbA1c, we did observe retinal neuroprotective effects [21]. Thus, although it cannot be excluded that a nonstatistically significant decrease in glycemia could influence the fate of diabetic retinopathy, it is unlikely since a strict control of the glycemia is required to limit retinopathy occurrence and severity in patients.

Our study also indicates that glibenclamide, particularly if formulated for ocular delivery, could be used for other degenerative retinal diseases associated with oxidative stress, inflammation and inflammasome activation, such as age-related macular degeneration (AMD). Recently, metformin was found to potentially reduce the incidence of AMD in large, retrospective studies [51] that need to be interpreted with caution [52]. Taking into account the fact that no treatment is currently available to prevent or delay retinal degeneration, the effects of old and known drugs with a known safety profile could be evaluated in treated patients that are affected by retinal diseases that induce rapid retinal cell death, such as retinal detachment [53].

## 5. Conclusions

This pilot study confirms our previous findings, indicating that the local intraocular delivery of glibenclamide was neuroprotective in several models of retinal damage. Further study is warranted to confirm these findings in other animal models and evaluate a correlation with human patients.

## Figures and Tables

**Table 1:** Primers references.

Gene	Primer Reference		
ABCC8	TaqMan™Gene Expression Assay (FAM) Rn01476317_m1	#4331182	Life Technologie SAS
GAPDH	TaqMan™Gene Expression Assay (FAM) Rn01775763_g1	#4331182	Life Technologie SAS

**Table 2:** Mean HbA1c at 2, 6 and 12 months in non-diabetic female, diabetic male and glibenclamide-treated male GK rats.

Non-Diabetic Females				Diabetic			Diabetic + GLI		
Age (Months)	Mean	SD	*n*	Mean	SD	*n*	Mean	SD	*n*
2	4.94	0.3	5	6.28	0.64	8	5.96	1.33	5
6	4.6	0.27	3	9.15	0.65	8	7.74	2.05	5
12	5	0.27	3	10.62	1.63	8	9.125	1.6	4

## Data Availability

Upon request.
